# Factors Associated with Burnout in Medical Academia: An Exploratory Analysis of Romanian and Moldavian Physicians

**DOI:** 10.3390/ijerph16132382

**Published:** 2019-07-04

**Authors:** Ovidiu Popa-Velea, Liliana Veronica Diaconescu, Iuliana Raluca Gheorghe, Oana Olariu, Iolanda Panaitiu, Mariana Cerniţanu, Ludmila Goma, Irina Nicov, Larisa Spinei

**Affiliations:** 1 Department of Medical Psychology, Faculty of Medicine, University of Medicine and Pharmacy “Carol Davila”, Bucharest 050474, Romania; 2 Department of Marketing and Medical Technology, Faculty of Medicine, University of Medicine and Pharmacy “Carol Davila”, Bucharest 050474, Romania; 3 Faculty of Medicine and Pharmacy, University “Dunărea de Jos”, Galaţi 800008, Romania; 4 Faculty of Medicine, University of Medicine and Pharmacy “Carol Davila”, Bucharest 050474, Romania; 5 Department of Management and Psychology, State University of Medicine and Pharmacy ”Nicolae Testemițanu”, Chişinău MD-2000, Moldova

**Keywords:** burnout, academic, physicians

## Abstract

This study aimed to assess the extent of burnout in Romanian and Moldavian academic physicians and to determine the predictive value of emotional intelligence (EI), coping strategies, work motivation (WM), perceived organizational support (POS), and the socio-demographic characteristics of burnout. Two hundred physicians (40% men, 60% women, mean age = 43.02, SD = 9.91) participated in the study. They were administered the Maslach Burnout Inventory−General Survey, Brief COPE Scale, Multidimensional Work Motivation Scale, Schutte’s Self-Report Emotional Intelligence Test, and Perceived Organizational Support Scale. Mann−Whitney U tests were used to assess the significance of intercountry differences, while hierarchical regressions were performed to investigate the predictive value of the independent variables on burnout. Moldavian participants had significantly lower scores in burnout and amotivation (*p* < 0.001) and higher scores in EI, POS, and WM (*p* < 0.001). The main burnout predictors were amotivation (β = 0.388, *p* < 0.001) and low POS (β = −0.313, *p*< 0.001) in Moldavian respondents, and WM (intrinsic: β = −0.620, *p* < 0.001; extrinsic: β = 0.406, *p* < 0.001) in Romanian participants. Moldavian respondents displayed better adjustment to academic stress. The distribution of burnout predictors suggests better sensitivity of respondents to organizational interventions in Moldova and to individual therapy in Romania. This data could serve to better tailor Public Health interventions addressing burnout in the academic environment.

## 1. Introduction

Burnout syndrome is commonly reported as an outcome of chronic occupational stress [1]. Among healthcare professionals, the overall burnout prevalence varies substantially, from 19% to 80.5% [2,3], depending on the type of measurement, timing, country and specialty [4,5,6,7,8]. Exploring the true extent of burnout and its predictors has become increasingly important in the last decades, considering the high societal costs [9] and redoubtable individual consequences, such as depression, anxiety, sleeping difficulties, poor job performance, lack of empathy, and aggressiveness [8,10,11,12,13,14]. In clinical settings, the most commonly acknowledged factors responsible for burnout are classified as being either organizational or individual. The first category comprises circumstances such as excessive workload [15], long working hours [7,8], or low perceived social support within the organization [7], while the second includes characteristics relevant for adjustment to demanding contexts, such as inefficient coping strategies and alexithymia [4,7,16,17].

For physicians who, in addition to their clinical responsibilities, have academic duties in universities, burnout risk factors become more specific within the aforementioned categories. Organizational factors may include, in this case, excessive working hours, devaluation of teaching work, inappropriate academic ranking [18], accumulation of functions beyond teaching [19,20], perceived publication pressure [19,21,22], or dissatisfaction regarding measures of scientific success [23]. In turn, individual contributors to burnout may encompass, in academic physicians, frustration at work [24,25], imbalance between personal and professional life [26] or passive coping strategies [27]. A series of personal characteristics such as hardiness [28], optimism [7], resilience [29,30], adaptive coping style [31], social support [32,33], emotional intelligence [34,35,36,37] and job motivation [38,39] may help in preventing or addressing burnout in these individuals. However, in order to produce notable beneficial results, these characteristics need to be stimulated and encouraged in the context of a competitive work climate in clinics and universities. The extent of this process, which demands early and systematic training, is not uniform across countries and educational systems.

Exposing burnout and its predictors in academic physicians is not an easy task, especially when taking into consideration the existence of numerous different methods to define and measure burnout, as well as the possible reluctance of respondents to report it. However, this process is undoubtedly necessary, as it may provide valuable, evidence-based cues for burnout prevention and offer concrete directions of intervention. In Romania and Moldova, two countries sharing a common cultural heritage, but having different health and education systems, this type of data is particularly valuable, since the study of burnout is at its beginnings, and there are limited financial resources to investigate it. In this respect, our research objective could fill a gap in the current literature and help better define Public Health policies designed to identify and address burnout. In particular, this study focused on several psychological variables (coping style, motivation, emotional intelligence), organizational variables (perceived organizational social support) and socio-demographic factors (age, gender, marital status, country of origin, work experience), with the purpose of investigating their relationship to burnout in Romanian and Moldavian physicians performing both clinical and educational work. The analysis comprised three distinct levels: (a) intercountry differences regarding burnout and the above-mentioned variables; (b) the search for relevant gender differences potentially related to burnout; and (c) measurement of the predictive value exerted by each of the study variables on burnout. Furthermore, given the fact that psychological and organizational variables have the potential to translate into Public Health strategies or psychotherapeutic interventions, while socio-demographic ones typically do not, this study aimed to offer relevant practical solutions for the prevention of burnout in academic physicians practicing in Romania and Moldova.

## 2. Materials and Methods

### 2.1. Participants

Academic physicians under the employment of “Carol Davila” University of Medicine and Pharmacy in Bucharest (Romania) and “Nicolae Testemițanu” University of Medicine and Pharmacy from Chişinău (Moldova), between the ages of 35 and 60 years, were considered for the study. Inclusion criteria required participants to complete all of the questionnaires, have at least 5 years of teaching experience, and no current somatic or psychiatric diseases.

Before taking part in the study, all participants completed written informed consent forms. The study was run in accordance with the World Medical Association Declaration of Helsinki and with the ethical guidelines published by the Committee of Ethics at the two participating institutions (ethical approval no. 7-9-2018). The study instruments were printed and administered in the presence of four researchers (OO, IRG, MC, IN), in case there were questions related to the process of filling them in. A total of 200 physicians (100 from Romania and 100 from Moldova) were included in the study (40% men, 60% women, mean age of 43.02, SD = 9.91). The overall response rate was 66.67%. The vast majority of participants had PhDs (68%) and a lower academic degree (44%), which was consistent with the proportion of academic degrees in the two universities taking part in the study. 

The majority of Romanian participants were women (72%). Fifty percent of respondents were married, their mean age was 42.86 (SD = 8.59), 87% had obtained a PhD, 42% held the academic title of lecturer, and mean academic experience was 12.27 years (SD = 8.28). Among Moldavians, the majority were males (52%), married (71%), with a mean age of 43.17 (SD = 11.12), 49% had obtained a PhD, 53% held the academic title of teaching assistant, and mean academic experience was 14.28 years (SD = 8.89).

### 2.2. Study Design

The study was cross-sectional and comprised a single administration of a series of psychometric instruments, in order to measure the participants’ perception of their working conditions and job satisfaction. Additionally, a set of socio-demographic data was collected during a preliminary structured interview, including information about gender, marital status, age and academic career (scientific degree, academic title, and academic experience).

(1) The Maslach Burnout Inventory−General Survey (MBI−GS) is a shortened form of the Maslach Burnout Inventory (MBI) [40], comprising 16 items, which are divided into three scales, similar to those from the original MBI scale emotional exhaustion (EE), cynicism (CY), and professional inefficacy (PI). The answers are given on a 7-point Likert scale (ranking from 0 = ”never” to 6 = “every day”). A high degree of burnout is reflected by high scores of exhaustion and cynicism and low professional efficacy scores. Although the original MBI measures burnout in human services occupations and one of its alternate forms (the MBI−Educators Survey) was developed to focus on teaching professions, in our study, we used the MBI−GS, because it has already been validated on a sample of Romanian healthcare professionals and, at the same time, has the advantage of shorter completion time. The Cronbach alpha of this version reaches 0.88 [41].
(2) The Brief COPE Scale contains 28 items, which measure 14 distinct coping strategies [42]. The answers are graded on a 4-point Likert scale from 1 = “I haven’t been doing this at all” to 4 = “I have been doing this a lot”. The test has generally been reported to have good reliability (0.50–0.90) [42], and the average value of Cronbach alpha coefficient was reported to be 0.70.
(3) The Multidimensional Work Motivation Scale (MWMS) assesses work motivation, within the theoretical framework offered by the self-determination theory [43]. The instrument contains 19 items, with answers graded on a 7-point Likert scale ranging from 1 = “not at all” to 7 = “completely”. It comprises the following five subscales: amotivation, external motivation (material and social), introjected motivation, motivation through identification, and intrinsic motivation. It is a cross-culturally validated scale, with good reported reliability (>0.70) [43].
(4) Schutte’s Self-Report Emotional Intelligence Test (SSEIT) measures perception, understanding, expression, regulating and harnessing of emotion in an individual’s self, as well as in others [44]. It contains 33 items with answers measured on a 5-point Likert scale, from 1 = “strongly disagree” to 5 = “strongly agree”. The total score is obtained by summing up the items’ scores; higher scores indicate more emotional intelligence. It also has good reliability (Cronbach alpha = 0.78) [44].
(5) Perceived Organizational Support Scale (POS) assesses possible feelings that individuals might have about the company (organization) for which they work [45]. We used the shorter version of the questionnaire (nine items), which is reported to display similar validity and reliability to the original one [45,46]. The answers were provided on a 7-point Likert scale (1 = “strongly disagree” to 7 = “strongly agree”). Higher scores indicate that respondents perceive their organization to be more supportive. The scale has good reliability (Cronbach alpha = 0.88).

### 2.3. Statistical Analysis

The collected data was processed and analyzed using the SPSS^®^ Statistics 20.0 software package.

The first phase of the analysis included a description of each study group, using statistical dispersion measures (mean and standard deviation) for quantitative variables, and contingency tables with frequencies for qualitative variables. In this phase, we also ran a set of Shapiro−Wilk normality tests for the quantitative variables. As their outcome was statistically significant, we concluded that the quantitative factors were not normally distributed, which oriented the choice of the statistical tests in the next step.

The second phase of the analysis consisted of running tests to evaluate the statistical significance of: differences in scores, by country, for all study variables (Mann–Whitney U test for independent samples);gender differences within each country (Mann−Whitney U test for independent samples); burnout predictors by performing a set of hierarchical regressions, designed to investigate the impact of the independent variables (coping strategies, work motivation, emotional intelligence, organizational support) on burnout.

The threshold for statistical significance for all tests was *p* < 0.05.

## 3. Results

### 3.1. Differences Among Study Groups

These differences were evaluated using Mann–Whitney U tests for independent samples (Table 1).

### 3.2. Gender Differences

The Mann−Whitney U test for independent samples was used to identify significant differences within the two study samples (Table 2).

### 3.3. Predictors of Burnout

In order to explore the impact of each study variable category on global burnout variance and its components, we conducted several hierarchical multiple regressions, using the stepwise method. For each burnout component, as well as for global burnout, the following set of regression models emerged (see Table 3, Table 4, Table 5 and Table 6).

#### 3.3.1. Emotional Exhaustion

In Moldavian physicians, EE was predicted by perceived organizational support (β = −0.318, t = −3.37, *p* < 0.001) in the first regression model, and in the second model by active coping (β = 0.208, t = 2.210, *p* < 0.02). The proportion of EE variance explained was 11% in model 1 and 15% in model 2.

In Romanian physicians, EE was predicted by intrinsic motivation in model 1 (β = −0.667, t = −8.863, *p* < 0.001), by intrinsic motivation (β = −0.638, t = −9.881, *p* < 0.001) and extrinsic motivation (β = 0.392, t = 6.071, *p* < 0.001) in model 2, and by intrinsic motivation (β = −0.627, t = −9.859, *p* < 0.001), extrinsic motivation (β = 0.337, t = 5.906, *p* < 0.001) and substance use (β= 0.138, t = 2.155, *p* < 0.03) in model 3. Forty-three percent of EE variance was explained by model 1, 58% by model 2, and 60% by model 3.

#### 3.3.2. Cynicism

In Moldavian respondents, CY was predicted by amotivation alone (β = 0.468, t = 5.238, *p* < 0.001) in model 1, by amotivation (β = 0.443, t = 5.202, *p* < 0.001) and the use of instrumental support (β = 0.290, t = 3.412, *p* < 0.001) in model 2, and by amotivation (β = 0.418, t = 5.021, *p* < 0.001), use of instrumental support (β = 0.325, t = 3.875, *p* < 0.001) and, inversely, by intrinsic motivation (β = −0.215, t = −2.559, *p* < 0.01) in model 3. The CY variance explained by the above-mentioned models was 21%, 28% and 32%, respectively.

In Romanian participants, CY was explained by five regression models. In model 1, 51% of CY variance was explained, inversely, by intrinsic motivation (β = −0.721, t = −10.297, *p*< 0.001). In model 2, 61% of CY variance was explained by intrinsic motivation (β = −0.697, t = −11.102, *p* < 0.001) and extrinsic motivation (β = 0.316, t = 5.037, *p* < 0.001). In model 3, 63% of CY variance was explained by intrinsic motivation (β = −0.642, t = −10.068, *p* < 0.001), extrinsic motivation (β = 0.270, t = 4.285, *p* < 0.001), and behavioral disengagement (β = 0.187, t = 2.828, *p* < 0.006). In model 4, 65% of CY variance was explained by intrinsic motivation (β = −0.603, t = −9.495, *p* < 0.001), extrinsic motivation (β = 0.324, t = 5.038, *p* < 0.001), behavioral disengagement (β = 0.200, t = 3.114, *p* < 0.002), and marital status (β = 0.170, t = 2.673, *p* < 0.009). Finally, in model 5, 67% of CY variance was explained by intrinsic motivation (β = −0.583, t = −9.222, *p* < 0.001), extrinsic motivation (β = 0.374, t = 5.533, *p* < 0.001), behavioral disengagement (β = 0.194, t = 3.069, *p* < 0.003), marital status (β = 0.162, t = 2.591, *p* < 0.011), and the use of instrumental support (β = −0.132, t = −2.086, *p* < 0.04).

#### 3.3.3. Personal Inefficacy

In Moldavian respondents, PI was predicted by amotivation (β = 0.542, t = 6.381, *p* < 0.001) in model 1, explaining 28% of its variance. In model 2, PI was predicted by amotivation (β = 0.459, t = 5.424, *p* < 0.001), and, inversely, by perceived organizational support (β = −0.281, t = −3.327, *p* < 0.001), together accounting for 35% of PI variance. In model 3, 40% of PI variance was explained by amotivation (β = 0.443, t = 5.437, *p* < 0.001), perceived organizational support (β = −0.254, t = −3.106, *p* < 0.002), and self-blame (β = 0.238, t = 3.035, *p* < 0.003).

In contrast, PI in Romanians was predicted by intrinsic motivation (β = −0.760, t = −11.593, *p* < 0.001) in model 1, by intrinsic motivation (β = −0.564, t = −7.848, *p* < 0.001) and by amotivation (β = 0.346, t = 4.816, *p* < 0.001) in model 2, and by intrinsic motivation (β = −0.505, t = −7.162, *p* < 0.001), amotivation (β = 0.388, t = 5.586, *p* < 0.001) and marital status (β= 0.196, t = 3.372, *p* < 0.001) in model 3. In model 4, PI was predicted by intrinsic motivation (β = −.559, t = −8.155, *p* < 0.001), amotivation (β = 0.243, t = 3.126, *p* < 0.002), marital status (β = 0.256, t = 4.438, *p* < 0.001) and extrinsic motivation (β = 0.234, t = 3.479, *p* < 0.004). In model 5, PI was predicted by intrinsic motivation (β = −0.513, t = −7.431, *p*< 0.001), amotivation (β = 0.245, t = 3.233, *p* < 0.002), marital status (β = 0.267, t = 4.747, *p* < 0.001), extrinsic motivation (β = 0.201, t = 3.012, *p* < 0.003) and behavioral disengagement (β = 0.415, t = 2.545, *p* < 0.013). In model 6, PI was predicted by intrinsic motivation (β = −0.515, t = −7.583, *p* < 0.001), amotivation (β = 0.214, t = 2.825, *p* < 0.0006), marital status (β = 0.281, t = 5.045, *p* < 0.001), extrinsic motivation (β = 0.249, t = 3.576, *p* < 0.001), behavioral disengagement (β = 0.129, t = 2.279, *p* < 0.025) and positive reframing (β = −0.112, t = −2.054, *p* < 0.043). Finally, in model 7, PI was predicted by intrinsic motivation (β = −0.551, t = −8.013, *p* < 0.001), amotivation (β = 0.196, t = 2.622, *p* < 0.001), marital status (β = 0.280, t = 5.107, *p* < 0.001), extrinsic motivation (β = 0.294, t = 4.111, *p* < 0.001), behavioral disengagement (β = 0.100, t = 1.766, *p*< 0.41), positive reframing (β = −0.129, t = −2.380, *p* < 0.019), and venting (β = 0.116, t = 2.131, *p* < 0.036). The above-mentioned predictors contributed to PI variance in a proportion of 57% (model 1), 65% (model 2), 68% (model 3), 71% (model 4), 73% (model 5), 74% (model 6), and 75% (model 7). 

#### 3.3.4. Global Burnout

The predictors of global burnout in Moldavian physicians were derived from three regression models. In model 1, global burnout was explained by amotivation (β = 0.481, t = 5.427, *p* < 0.001), and in model 2 by amotivation (β = 0.388, t = 4.436, *p* < 0.001) and, inversely, by perceived organizational support (β = −0.313, t = −3.577, *p* < 0.001). In model 3, global burnout was predicted by amotivation (β = 0.402, t = 4.714, *p* < 0.001), acceptance (β = 0.208, t = 2.547, *p* < 0.012), and, inversely by perceived organizational support (β = −0.308, t = −3.619, *p* < 0.001). These variables explained 22% of variance in global burnout in model 1, 30% in model 2, and 34% in model 3.

Global burnout in Romanians was predicted by intrinsic motivation (β = −0.763, t = −11.321, *p*< 0.001) in model 1, explaining 56% of the variance. In model 2, it was predicted by intrinsic motivation (β = −0.728, t = −12.545, *p* < 0.001) and extrinsic motivation (β = 0.330, t = 5.677, *p* < 0.001), explaining 66% of the variance. In model 3, global burnout was predicted by intrinsic motivation (β = −0.688, t = −12.140, *p* < 0.001), extrinsic motivation (β = 0.396, t = 6.728, *p* < 0.001) and marital status (β = 0.197, t = 3.295, *p* < 0.001), explaining 69% of the variance. In model 4, global burnout was predicted by intrinsic motivation (β = −0.638, t = −11.034, *p* < 0.001), extrinsic motivation (β = 0.361, t = 6.159, *p* < 0.001), marital status (β = 0.209, t = 3.601, *p* < 0.001) and behavioral disengagement (β = 0.159, t = 2.710, *p* < 0.008). Finally, in model 5, the predictors of global burnout were intrinsic motivation (β = −0.620, t = −10.767, *p* < 0.001), extrinsic motivation (β = 0.406, t = 6.588, *p* < 0.001), marital status (β = 0.202, t = 3.534, *p* < 0.001), behavioral disengagement (β = 0.153, t = 2.657, *p* < 0.001) and use of instrumental support (β=−0.119, t = −2.064, *p* < 0.042). The predictors of model 4 explained 71% of the global burnout variance, while the predictors of model 5 explained 72%.

## 4. Discussion

Regarding the first objective of the study (inventorying significant inter-country differences in the study variables), Moldavian participants obtained better scores than Romanian participants in all three components of burnout, as well as global burnout, and in intrinsic motivation (through introjection, identification), but also extrinsic motivation. In contrast, the Romanian participants had significantly higher scores in amotivation, suggesting their incapacity to access and mobilize inner resources to cope (especially over a longer period of time) in a stressful work environment.

Emotional intelligence may have had an indirect influence on the low burnout scores in Moldavian participants, via (a) the positive perception of academic life and its challenges [47]; and (b) the general positive influence of emotional intelligence on the academic institution per se, in turn benefiting its employees [48,49]. It is important to note that, beyond its potential role in helping academic physicians to overcome their personal difficulties, emotional intelligence could be passed on to students through modeling, thereby representing a useful tool in preventing burnout throughout the academic community [49].

Such useful tools are: organizational social support (high scores have already been reported in the literature as playing a moderating role when confronting work stress, in health care professionals) [50]; and functional coping strategies (e.g., active coping, positive reframing, acceptance).

In contrast, Romanian respondents had higher scores in dysfunctional coping strategies (e.g., denial, substance use, behavioral disengagement) and reported significantly more frequent use of emotional social support. Concerning the latter, taking into account the highly prevalent rate of burnout in this population, it seems that this potentially valuable strategy did not have the desired protective effect.

Concerning gender differences, they were much more substantial among Romanian physicians; women included in the study had statistically higher scores compared to men, for intrinsic and extrinsic motivation, the use of social support (be it emotional or instrumental), positive reframing, planning and religious coping. Virtually, all of these variables have a buffering effect against stress, suggesting that female academic physicians in Romania may benefit from a wider array of tools to fight or prevent burnout, compared to men. In contrast, the only significant gender difference identified in Moldavian respondents was the use of acceptance as a coping mechanism, which was more frequent in women, and appears to be consistent with the gender stereotypes present in Moldavian society [51].

Burnout predictors in Moldavian participants were comprised of amotivation (for CY, PI and global burnout), low perceived organizational social support (for EE, PI, and global burnout), and the use of instrumental social support (for CY). While the direct predictive relationship between a lack of motivation and burnout is consistently reported in literature [52,53,54], the importance of social support (in its organizational and instrumental dimensions) on burnout appears noteworthy, especially with regards to its ambivalent nature (with organizational support playing a protective role and instrumental support having an opposite effect). These results may originate from a particularity of the Moldavian academic system, which historically represents a continuation of the hyper-centralized Soviet model of education. In this type of system, support is often expected to be provided by a formal structure rather than by an individual, with the former often valued substantially more, in terms of predictability and continuity. In terms of potential interventions to prevent or address burnout, the above data suggests that group interventions may be more effective or at least more easily accepted by Moldavian physicians facing work challenges in the academic environment.

In Romanian physicians, burnout predictors included motivation, in all its forms: intrinsic (EE, CY, PI, and global score), extrinsic (EE, CY, and global score) and amotivation (PI). As one would expect, extrinsic motivation and amotivation favored burnout, while intrinsic motivation played a protective role. The high predictive value of motivation, compared to, for example, social support, which had no predictive value relating to burnout, raises the question of whether the latter is an aspect which many Romanian health care professionals working in the academic environment perceive as futile or inexistent. In the past three decades, there have been numerous changes in the academic landscape, not least of all, changes related to the professional roles of academic employees. Throughout this complicated and prolonged process, many medical academic professionals may have reached the conclusion that they should not expect too much support from any kind of organization designed or functioning on a hierarchical basis. This phenomenon may be part of a more general state of atomization of Romanian society [55], with significant underlying costs, in terms of solidarity and cohesion of the social fabric. Despite this rather pessimistic outlook, the data may be informative relating to the potential effectiveness of interventions designed to prevent burnout; individual counseling and therapy seem preferable in the current state of evolution of Romanian society. 

As a whole, in terms of categories of burnout predictors, socio-demographic factors play a minor role in both countries, in contrast to psychological determinants (in Romania) and organizational variables (in Moldova). Relating to practical interventions designed to prevent or address burnout, this finding reveals a silver lining, as the last two categories of factors can be approached individually, via psychotherapy or counseling, or at the organizational level.

This study has several limitations which can affect the generalizability of its results. The cross-sectional design offers only a snapshot of burnout and its determinants in the academic context and provides no information about its dynamics. From this perspective, a larger prospective multicentric study could offer valuable information for the design of interventions targeting burnout in medical academia. The gender ratio of study participants across the two studied countries was asymmetric, possibly due to differences in equal opportunity policies and perceived attractiveness of academic careers in Romania and Moldova. Additional factors, such as understanding of study questions or social desirability may have also influenced the study responses. 

## 5. Conclusions

The results of this study offer important information about the current state of burnout in academic physicians practicing in Romania and Moldova, and about the categories of predictors involved in its occurrence. This, in its turn, may serve to prioritize certain directions of action designed to prevent burnout in medical professionals working in an academic environment, to increase the sensitivity of academic communities towards this topic, and, just as importantly, to ensure a better chance for the provision of high quality education for medical students.

## Figures and Tables

**Table 1:** Differences between Moldavian and Romanian participants.

Variable	Mean (SD)		Mann−Whitney U Test	*p*
	Moldova	Romania		
Exhaustion	9.79 (7.02)	20.96 (6.29)	1318.50	0.001
Cynicism	8.51 (6.71)	23.14 (8.11)	926.500	0.001
Personal inefficacy	5.30 (5.59)	21.57 (8.54)	677.500	0.001
Global burnout	21.72 (16.04)	61.65 (20.39)	750.000	0.001
Amotivation	1.43 (0.75)	3.02 (1.74)	1949.50	0.001
Motivation through introjection	5.04 (1.50)	3.73 (1.70)	2841.000	0.001
Motivation through identification	5.55 (1.46)	4.04 (1.89)	2696.000	0.001
Intrinsic motivation	5.62 (1.46)	3.80 (1.78)	2157.50	0.001
Emotional intelligence	125.38 (14.14)	100.46 (27.10)	2176.00	0.001
Organizational support	42.82 (11.64)	19.85 (8.78)	634.50	0.001
Active coping	5.82 (1.76)	4.59 (1.46)	2991.00	0.001
Denial	3.24 (1.43)	4.61 (1.72)	2693.50	0.001
Substance use	2.56 (1.08)	3.84 (1.57)	2697.00	0.001
Use of emotional support	4.42 (1.42)	4.92 (1.80)	4085.00	0.02
Behavioral disengagement	3.28 (1.59)	4.86 (1.63)	2298.00	0.001
Positive reframing	5.35 (1.62)	4.84 (1.13)	3947.00	0.008
Planning	6.55 (1.53)	4.94 (1.55)	2326.00	0.001
Acceptance	5.92 (1.66)	5.07 (1.53)	3532.50	0.001

Moldavian respondents had significantly lower scores in burnout and all its components, as well as amotivation, and higher scores in emotional intelligence, perceived organizational support and extrinsic and intrinsic motivation, compared to Romanian participants. Preferred coping strategies included denial, substance use, behavioral disengagement and the use of emotional support in Romanians; in contrast, Moldavians predominantly relied on active coping, positive reframing, planning and acceptance.

**Table 2:** Gender differences in Moldova and Romania.

Country	Variable	Gender		Mann−Whitney U Test	*p*
		Male	Female		
Moldova	Acceptance	44.56	56.94	939.000	0.03
Romania	Extrinsic motivation	40.77	54.28	735.500	0.03
	Introjection	38.45	55.19	670.500	0.009
	Identification	40.13	54.53	717.500	0.025
	Use of emotional support	37.18	55.68	635.000	0.004
	Use of instrumental support	37.46	55.57	643.000	0.004
	Positive reframing	38.39	55.21	669.000	0.007
	Planning	39.95	54.60	712.500	0.020
	Religion	41.23	54.10	748.500	0.040

The gender differences were much more significant in Romanian respondents, with higher scores obtained by women in intrinsic and extrinsic motivation, use of emotional support, use of instrumental support, positive reframing, planning and religious coping. In contrast, Moldavian gender differences were restricted to acceptance, where women had significantly higher scores than men.

**Table 3:** Hierarchical linear regression models of emotional exhaustion in Moldova and Romania.

Country	Models Predictors	R	R^2^	Adj. R^2^	SEE^c^	R^2^ Change	df1	df2	F Change	Sig.
MDA^a^	1:(c.), POS^d^	0.354	0.126	0.117	6.606	0.126	1	98	14.07	0.001
	2:(c.), POS^d^, Active coping	0.409	0.168	0.150	6.479	0.042	1	97	4.883	0.029
ROU^b^	1:(c.), IM^e^	0.667	0.445	0.439	4.713	0.445	1	98	78.55	0.001
	2:(c.), IM^e^, EM^f^	0.773	0.598	0.589	4.033	0.153	1	97	36.85	0.001
	3: (c.), IM^e^, EM^f^, Substance Use	0.785	0.616	0.604	3.959	0.019	1	96	4.644	0.034

^a^MDA = Moldova; ^b^ROU = Romania; ^c^SEE = standard error of the estimate; ^d^POS = Perceived organizational support; ^e^IM = Intrinsic Motivation; ^f^EM = Extrinsic Motivation; (c.) = constant.

**Table 4:** Hierarchical linear regression models of cynicism in Moldova and Romania.

Country	Models Predictors	R	R^2^	Adj. R^2^	SEE^c^	R^2^ Change	df1	df2	F Change	Sig.
MDA^a^	1: (c.), AM^d^	0.468	0.219	0.211	5.966	0.219	1	98	27.440	0.001
	2: (c.), AM^d^, Use of instrumental support	0.550	0.302	0.288	5.666	0.084	1	97	11.639	0.001
	3: (c.), AM^d^, Use of instrumental support, IM^e^	0.589	0.347	0.327	5.511	0.045	1	96	6.549	0.012
ROU^b^	1: (c.), IM^e^	0.721	0.520	0.515	5.656	0.520	1	98	106.01	0.001
	2: (c.), IM^e^, EM^f^	0.787	0.619	0.611	5.061	0.100	1	97	25.372	0.001
	3: (c.), IM^e^, EM^f^, Behavioral Disengagement	0.805	0.649	0.638	4.888	0.029	1	96	7.996	0.006
	4: (c.), IM^e^, EM^f^, Behavioral Disengagement, Marital Status	0.820	0.673	0.659	4.739	0.025	1	95	7.145	0.009
	5: (c.), IM^e^, EM^f^, Behavioral Disengagement, Marital Status, Use of Instrumental Support	0.829	0.688	0.671	4.657	0.014	1	94	4.351	0.040

^a^MDA = Moldova; ^b^ROU = Romania; ^c^SEE = standard error of the estimate; ^d^AM = amotivation; ^e^IM = intrinsic motivation; ^f^EM = extrinsic motivation; (c.) = constant.

**Table 5:** Hierarchical linear regression models of personal inefficacy in Moldova and Romania.

Country	Predictors	R	R^2^	Adj. R^2^	SEE^c^	R^2^ Change	df1	df2	F Change	Sig.
MDA^a^	1: (c.), AM^d^	0.542	0.294	0.286	4.727	0.294	1	98	40.720	0.001
	2: (c.), AM^d^, POS^e^	0.605	0.366	0.353	4.501	0.072	1	97	11.070	0.001
	3:(c.), AM^d^, POS^e^, Self-blame	0.649	0.421	0.403	4.322	0.056	1	96	9.213	0.003
ROU^b^	1: (c.), IM^f^	0.760	0.578	0.574	5.575	0.578	1	98	134.39	0.001
	2: (c.), IM^f^, AM^d^	0.812	0.660	0.653	5.034	0.081	1	97	23.195	0.001
	3: (c.), IM^f^, AM^d^, Marital Status	0.834	0.696	0.686	4.785	0.036	1	96	11.370	0.001
	4: (c.), IM^f^, AM^d^, Marital Status, EM^g^	0.854	0.730	0.719	4.530	0.034	1	95	12.103	0.001
	5: (c.), IM^f^, AM^d^, Marital Status, EM^g^, Behavioral Disengagement	0.865	0.747	0.734	4.405	0.017	1	94	6.476	0.013
	6: (c.), IM^f^, AM^d^, Marital Status, EM^g^, Behavioral Disengagement, Positive reframing	0.871	0.758	0.743	4.331	0.011	1	93	4.218	0.043
	7: (c.), IM^f^, AM^d^, Marital Status, EM^g^, Behavioral Disengagement, Positive reframing, Venting	0.877	0.770	0.752	4.251	0.011	1	92	4.541	0.036

^a^MDA = Moldova; ^b^ROU = Romania; ^c^SEE = standard error of the estimate; ^d^AM = amotivation; ^e^POS = perceived organizational support; ^f^IM = intrinsic motivation; ^g^EM = extrinsic motivation; (c.) = constant.

**Table 6:** Hierarchical linear regression models of global burnout in Moldova and Romania.

Country	ModelsPredictors	R	R^2^	Adj. R^2^	SEE^c^	R^2^ Change	df1	df2	F Change	Sig.
MDA^a^	1: (c.), AM^d^	0.481	0.231	0.223	14.141	0.231	1	98	29.449	0.001
	2:(c.), AM^d^,POS^e^	0.566	0.321	0.307	13.360	0.090	1	97	12.792	0.001
	3:(c.), AM^d^, POS^e^, Acceptance	0.603	0.364	0.344	12.997	0.043	1	96	6.488	0.012
ROU^b^	1: (c.), IM	0.753	0.567	0.562	13.626	0.567	1	98	128.15	0.001
	2:(c.), IM^f^, EM^g^	0.821	0.675	0.668	11.866	0.108	1	97	32.234	0.001
	3:(c.), IM^f^, EM^g^, Marital Status	0.841	0.708	0.699	11.305	0.033	1	96	10.859	0.001
	4:(c.), IM^f^, EM^g^, Marital Status, Behavioral disengagement	0.854	0.729	0.717	10.949	0.021	1	95	7.342	0.008
	5: (c.), IM^f^, EM^g^, Marital Status, Behavioral disengagement, Use of instrumental support	0.861	0.741	0.727	10.766	0.012	1	94	4.261	0.042

^a^MDA = Moldova; ^b^ROU = Romania; ^c^SEE = standard error of the estimate; ^d^AM = amotivation; ^e^POS = perceived organizational support; ^f^IM = intrinsic motivation; ^g^EM = extrinsic motivation, (c.) = constant.
